# HIV prevalence, incidence and risk behaviours among men who have sex with men in Yangzhou and Guangzhou, China: a cohort study

**DOI:** 10.7448/IAS.17.1.18849

**Published:** 2014-08-06

**Authors:** Qian-Qiu Wang, Xiang-Sheng Chen, Yue-Ping Yin, Guo-Jun Liang, Rui-Li Zhang, Ning Jiang, Xi-Ping Huan, Bin Yang, Qiao Liu, Yu-Jiao Zhou, Bao-Xi Wang

**Affiliations:** 1 National Center for STD Control and Prevention, China Centers for Diseases Control and Prevention, Institute of Dermatology, Chinese Academy of Medical Sciences, Nanjing, China; 2 Jiangsu Provincial Centers for Disease Control and Prevention, Nanjing, China; 3 Guangdong Provincial Center for Skin Diseases and STD Control, Guangzhou, China; 4 Hainan Provincial Center for Skin Diseases and STD Control, Haikou, China; 5 Guangxi Centers for Disease Control and Prevention, Nanning, China

**Keywords:** HIV, prevalence, incidence, risk behaviours, men who have sex with men, Yangzhou, Guangzhou

## Abstract

**Introduction:**

In China, the prevalence and incidence of HIV among men who have sex with men (MSM) in large-sized cities have drawn much attention. In contrast, there has been a paucity of research focussing on the sexual health of MSM of medium-sized cities. This study fills this important gap in the knowledge by investigating the sexual health of MSM in a medium-sized city (Yangzhou) and a large-sized city (Guangzhou).

**Methods:**

A baseline survey and a prospective cohort study were conducted among MSM in Yangzhou and Guangzhou from July 2009 to September 2010. A total of 622 MSM (317 from Yangzhou and 305 from Guangzhou) were screened for eligibility. Prevalence and incidence of HIV infection, as well as its risk factors, were investigated.

**Results:**

Baseline HIV prevalence was 14.5%, and overall HIV incidence density was 6.78 per 100 person-years (PY) among Yangzhou MSM. Risk factors for HIV prevalence that were significant in multivariate models were older age, married status, unprotected sex with female partners, sexually transmitted disease (STD)-associated symptoms and syphilis positivity. Risk factors for HIV incidence that were significant in multivariate models were STD-associated symptom and syphilis positivity. Compared to Yangzhou MSM, Guangzhou MSM had a lower HIV prevalence (6.2%; *p*<0.05) and lower overall HIV incidence density (5.77 per 100 PY). Risk factors for HIV prevalence that were significant in multivariate models were married status, unprotected anal sex with men and syphilis positivity. The single risk factor for HIV incidence that was significant in multivariate models was unprotected anal sex with men.

**Conclusions:**

This study showed a high prevalence and incidence of HIV among Yangzhou MSM, which suggest a more serious HIV epidemic than that in large-sized cities. Further investigation targeting MSM in medium-sized cites is urgently needed to prevent the spread of the HIV epidemic in China.

## Introduction

In China, an estimated 780,000 people were living with HIV at the end of 2011, according to the latest estimation conducted jointly by the Ministry of Health of China, World Health Organization and Joint United Nations Programme on HIV/AIDS (UNAIDS) in 2011 [1]. Injecting drug users, female sex workers and their clients, and men who have sex with men (MSM) were identified as the most high-risk populations contributing to the HIV epidemic in China. In view of the large population size of MSM, as well as high HIV prevalence and increasing incidence rates, homosexual transmission has overtaken injection drug use as the dominant route of HIV infections in China [2–5].

Surveillance data in 2011 demonstrated an overall HIV prevalence of 6.3%, and 24 of 105 sentinel sites showed HIV prevalence rates among MSM that were higher than 10% [6]. However, these studies are concentrated in large-sized cities and have included little data from medium-sized cities. As a result of the urbanization in China and fewer opportunities being provided by large-sized cities, the floating population size is sharply increasing in medium-sized cities. The behavioural characteristics and spatial transition of the floating population are important factors in HIV spread [7]. Meanwhile, economic development and open-mindedness also brought about the increase of MSM populations in medium-sized cities. However, limited epidemiological and behavioural data are available; further studies are needed that focus on the MSM population in medium-sized cities.

Yangzhou is a city located in the centre of Jiangsu Province, eastern China, with a total population of 4.6 million. Since the first AIDS case was found in 1997, the number of HIV infections increased significantly to 256, with an average annual growth rate of 27.1% by the end of December 2010 [8]. In recent years, sexual transmission has become the major route of HIV transmission in the city, increasing from 36.9% in 2007 to 91.2% in 2010. In China, a total of 48,000 people were newly diagnosed with HIV in 2011; 81.6% were infected by sexual transmission, among whom 29.4% were infected by homosexual transmission [1]. Thus, we assume that MSM and HIV-positive MSM must be important factors contributing to the HIV epidemic. However, little is known about the demographic characteristics and HIV risk factors of MSM in this city. Guangzhou, the capital of Guangdong Province in southern China, has a total population of 12.7 million, and it has an accepting and diverse culture with numerous immigrants and greater chances of employment, which attracts MSM from all over China. The MSM HIV prevalence in Guangzhou rapidly increased in recent years. The observed HIV prevalence increased from 1.3% in 2006 to 5.8% in 2011 based on sentinel surveillance among MSM [1,9–12].

In order to have a complete understanding of the HIV prevalence, incidence and risk factors of MSM in Yangzhou, a prospective cohort study was conducted. We chose Guangzhou MSM as a comparison sample to explore differences between MSM in Yangzhou and in Guangzhou. This study will, therefore, provide evidence for formulating targeted measures and preventing the HIV epidemic among MSM in medium-sized cities.

## Methods

### Participants

A cohort study was conducted among MSM in Yangzhou and Guangzhou from July 2009 to September 2010 with a frequency of three months between visits. Participants were those who met the following criteria: 18 years of age or older, male, had any sexual experience (oral sex, anal sex or mutual masturbation) with another man in the past 12 months, were able to provide written informed consent and were willing to participate in the 12-month follow-up survey. Baseline data were collected from all participants, including those who tested positive for syphilis and/or HIV; however, only HIV-negative participants were enrolled in the cohort.

### Study procedures

The snowball sampling method was used to recruit participants in Yangzhou and Guangzhou. The sample size was estimated based on the prevalence of HIV in 2008, which was 4.62% among Yangzhou MSM and 5.20% among Guangzhou MSM (unpublished data, HIV sentinel surveillance, National Center for STD Control, China CDC, 2008); 352 subjects (Yangzhou) and 328 subjects (Guangzhou) were required. The participants were recruited from MSM venues such as bars, Internet chat rooms, clubs, tea bars, public baths and sauna houses, parks and public restrooms. Initial “seed” participants were recommended by gay organizations and staff of MSM activity venues. Each initial seed was invited to participate in the study and then asked to provide another person with the same inclusion criteria as a new seed. The process continued in the same way until a sufficient number of subjects was obtained.

At the beginning of the survey, the interviewer, who was trained in techniques for conducting research, briefly introduced the purpose and procedures of the study, and then participants were asked to complete the questionnaire by themselves. After completing the questionnaire, the participants were asked to draw 5–7 ml of venous blood for HIV and syphilis tests. Interviews were once again conducted, and blood samples were obtained and tested for HIV and syphilis, every three months. The interviews occurred in a private room to ensure privacy. Written informed consent was obtained from every participant before the baseline interview and tests. All participants were provided with condoms and lubricant after completing the questionnaire. General knowledge about syphilis and HIV and information on how to practice safe sexual behaviour during the course of the pre- and post-test were also provided. Those who tested positive for syphilis and/or HIV during the baseline screening survey and during the cohort period were subsequently referred for medical management at local designated medical centres. This study was approved by the Ethics Committee of the National Center for STD Control, China CDC.

### Data collection

Data were collected through structured questionnaire-based interviews providing socio-demographic characteristics, drug use, sexual health services, sexually transmitted disease (STD) history, STD-associated symptoms and sexual behaviour information. Participants’ questionnaires were confidential but not anonymous, and they were linked to the follow-up diagnostic data.

### Laboratory testing

Sera separated from the venous blood of each participant were tested for HIV and syphilis antibodies. The screening test algorithm for the HIV antibody was tested using the enzyme-linked immunosorbent assay (ELISA; Wansheng Biotech Inc., Beijing, China), and positive tests were confirmed by the Western blot assay (HIV Blot 2.2, Genelabs Diagnostics, Singapore). The toluidine red unheated serum test (TRUST; Rongsheng Biotech Inc., Shanghai, China) was used for syphilis screening and quantitative analysis. The *Treponema pallidum* particle agglutination test (TPPA; Fujirebio Inc., Tokyo, Japan) or *Treponema pallidum*–ELISA (TP-ELISA, Wansheng Biotech Inc., Beijing, China) was used for syphilis test confirmation. Subjects with plasma positive for both TPPA and TRUST were defined as being currently infected with syphilis. In addition, baseline TRUST-negative cases that turned positive for both TPPA and TRUST were defined as syphilis seroconversion during the follow-up period. Baseline TPPA-negative cases that turned TPPA positive (regardless of TRUST status) at follow-up were also considered as syphilis seroconversion. Likewise, baseline HIV-negative cases that turned positive, as confirmed by the Western blot assay, were defined as HIV seroconversion during the follow-up period.

### Statistical analysis

Data were double entered and then checked for accuracy using Epi Data software (version 3.0; Epi Data Association, Odense, Denmark). HIV incidence density was calculated based on the Poisson distribution, with person-years (PY) of follow-up as the denominator. Odds ratios (ORs) and 95% confidence intervals (CIs) were calculated. Bivariate logistic regression and multivariate logistic regression were performed to adjust ORs for potential confounding factors. Continuous variables were described using means and standard deviations, and categorical data were described and analysed using frequencies and *χ* ^2^ tests. Only variables that were significant in bivariate analyses at *p*<0.1 were included in multivariate logistic regression models. *P* values less than 0.05 (two tailed) were considered statistically significant. All statistical analyses were conducted using the Statistical Package for Social Sciences software (SPSS15.01™; SPSS Inc., Chicago, IL, USA).

## Results

### Demographic characteristics

In this study, a total of 622 MSM (317 from Yangzhou and 305 from Guangzhou) were eligible for attending a baseline screening survey, and 65 (46 from Yangzhou and 19 from Guangzhou) were HIV positive and therefore excluded from the cohort study. A total of 557 HIV-negative MSM were included in the 12-month follow-up cohort.

In the baseline survey, there were significant differences in demographic characteristics between Yangzhou MSM and Guangzhou MSM. In the Yangzhou sample, 65.9% were between 20 and 40 years old (mean age: 36.1±10.8), whereas 92.1% of the Guangzhou sample participants were between 20 and 40 years old (mean age: 21.1±6.9). Compared to Guangzhou MSM, Yangzhou MSM were significantly older (*p*<0.01). Nearly two-thirds (59.0%) of Yangzhou MSM were married, but the majority of Guangzhou MSM (78.4%) were single. The most popular venue to find male sexual partners for the Yangzhou sample was the public bathhouse (53.6%), followed by bars (20.8%) and the Internet (19.6%), whereas the Guangzhou sample mostly used the Internet (79.0%). Guangzhou MSM had received more sexual health services and were more likely to have been diagnosed with an STD (all *p*<0.01) than Yangzhou MSM (Table 1).

**Table 1:** Comparison of baseline demographic characteristics among MSM in Yangzhou and Guangzhou, China

	Yangzhou		Guangzhou			
			
Characteristic	*n*	%	*n*	%	Adjusted χ^2^	*p*
Age (years)					70.17	<0.001
<20	5	1.6	6	2.0		
20–40	209	65.9	281	92.1		
>40	103	32.5	18	5.9		
Education level					134.77	<0.001
Middle school or lower	110	34.7	26	8.6		
High school	135	42.6	73	23.9		
College or higher	72	22.7	206	67.5		
Marital status					111.62	<0.001
Single	118	37.2	239	78.4		
Married	187	59.0	56	18.3		
Divorced	12	3.8	10	3.3		
Ethnicity					1.13	0.287
Han	311	98.1	294	96.4		
Other	6	1.9	11	3.6		
Sexual orientation					11.29	<0.001
Homosexual	134	42.3	171	56.0		
Bisexual or heterosexual	183	57.7	134	44.0		
Venue where sex is found					321.29	<0.001
Bathhouse	170	53.6	5	1.6		
Bar	66	20.8	8	2.6		
Internet	62	19.6	241	79.0		
Other	19	6.0	51	16.6		
Commercial sex in the past 6 months					4.33	0.037
Yes	29	9.1	14	4.6		
No	288	90.9	291	95.4		
Condom use during last commercial sex encounter					0.05	0.812
Yes	17	58.6	8	57.1		
No	12	41.4	6	42.9		
Anal sex with men in the past 6 months					1.88	0.170
Yes	202	63.7	177	58.0		
No	115	36.3	128	42.0		
Condom use during last anal sex with a man					6.12	0.013
Yes	140	69.3	100	74.8		
No	62	30.7	77	25.2		
Sex with female partners in the past 6 months					115.34	<0.001
Yes	188	59.3	52	17.0		
No	129	40.7	253	83.0		
Condom use during last sexual encounter with female partner					0.05	0.818
Yes	56	29.8	14	26.9		
No	132	70.2	38	73.1		
Illegal drug use in the past 12 months					0.06	0.796
Yes	6	1.9	4	1.3		
No	311	98.1	301	98.7		
STD-associated symptoms in the past 12 months					2.13	0.144
Yes	28	8.8	39	12.8		
No	289	91.2	266	87.2		
Having been diagnosed with an STD in the past 12 months					30.78	<0.001
Yes	30	9.5	82	26.9		
No	287	90.5	223	73.1		
Getting sexual health services in the past 12 months					73.84	<0.001
Yes	175	55.2	265	86.9		
No	142	44.8	40	13.1		
Syphilis positive					168.6	<0.001
Yes	98	30.9	20	6.5		
No	219	69.1	285	93.5		
HIV positive					10.52	<0.001
Yes	46	14.5	19	6.2		
No	271	85.4	286	93.8		

STD: sexually transmitted disease.

### Sexual behaviours and condom use

In this investigation, sexual behaviours and condom use were also different between Yangzhou MSM and Guangzhou MSM. The proportion of Yangzhou MSM who had sexual intercourse with females was three times higher than in the Guangzhou sample (59.3 and 17.0%, respectively; *p*<0.01), but the percentages of condom use during the last intercourse with female partners were not different between the two groups (*p*>0.05). No differences were observed between the two groups regarding condom use during both the last commercial sex act and anal sex with a man in the past six months (all *p*>0.05). However, of those who had anal sex with a man in the past six months, Yangzhou MSM were less likely to use condoms during their last sexual act than Guangzhou MSM (69.3% vs. 74.8%; *p*<0.05) (Table 1).

### Prevalence of HIV and syphilis, and associated risk factors of HIV prevalence

In Yangzhou MSM, the baseline prevalence of HIV and syphilis was 14.5% and 30.9%, respectively. The prevalence of HIV and syphilis was significantly higher in Yangzhou MSM than in Guangzhou MSM (6.2% and 6.5%; both *p*<0.01), indicating a higher risk of HIV infection. The associated risk factors for HIV infection were assessed by logistic regression. In the multivariate analysis, risk factors of HIV prevalence were associated with older age (older than 40 years), married status, sex with females, unprotected sex with females and syphilis positivity. For the Guangzhou sample, risk factors that were significant in the multivariate logistic regression analysis predicting HIV prevalence were married status, divorced status, anal sex with a man, unprotected anal sex with a man and syphilis positivity (Table 2) (Appendix Table 1).

**Table 2:** Factors associated with HIV prevalence at baseline screening among MSM in Yangzhou and Guangzhou, China

	Yangzhou Multivariate analysis			Guangzhou Multivariate analysis		
	
Factors	*n*	OR (95% CI)	*p*	*n*	OR (95% CI)	*p*
Age (years)						
20–40	21 (209)	1		16 (281)	–	
>40	25 (103)	4.34 (2.25–8.36)	<0.001	3 (18)	–	
Marital status						
Single	9 (118)	1		9 (239)	1	
Married	36 (187)	2.88 (1.33–6.24)	0.007	8 (56)	13.48 (2.06–88.18)	0.007
Divorced	1 (12)	–		2 (10)	5.84 (1.91–17.80)	0.002
Anal sex with men in the past 6 months						
Yes	30 (202)	–		17 (177)	9.03 (1.85–44.10)	0.007
No	16 (115)	–		2 (128)	1	
Condom use during last anal sex with a man						
Yes	21 (40)	–		5 (110)	1	
No	9 (62)	–		12 (67)	4.93 (1.65–4.69)	0.004
Sex with female partners in the past 6 months						
Yes	36 (188)	2.50 (1.14–5.48)	0.022	3 (52)	–	
No	10 (129)			16 (253)	–	
Condom use during last sexual encounter with a female partner						
Yes	3 (56)	1		1 (14)	–	
No	33 (132)	5.89 (1.73–20.11)	0.005	2 (38)	–	
Syphilis positive						
Yes	26 (98)	3.47 (1.76–6.84)	<0.001	5 (20)	6.29 (1.63–24.29)	0.008
No	20 (219)	1		14 (285)	1	

CI: confidence interval; OR: odd ratio.

### Incidence of HIV and syphilis, and associated risk factors of HIV seroconversion

Of 557 HIV-negative MSM who were retained in the cohort, 23 cases of HIV infection (11 in Yangzhou and 12 in Guangzhou, respectively) were detected during the 12-month follow-up. The HIV incidence density was calculated to be 6.78/100 PY (Yangzhou) and 5.77/100 PY (Guangzhou), respectively. Among the retained 344 MSM who were seronegative for syphilis at baseline, a total of 43 cases of syphilis seroconversion were observed (34 in Yangzhou and 10 in Guangzhou), resulting in an incidence density of 29.82/100 PY in Yangzhou and 5.33/100 PY in Guangzhou. The associated risk factors of HIV seroconversion were defined in multivariate regression analyses. In multivariate analysis, married status (OR: 0.63; 95% CI: 1.29–53.00), unprotected sex with female partners in the past six months (OR: 11.11; 95% CI: 1.19–80.24) and syphilis positivity (OR: 5.13; 95% CI: 1.30–20.20) were found to be significantly associated with HIV seroconversion in the Yangzhou sample. In the Guangzhou sample, anal sex with men in the past six months (OR: 9.16; 95% CI: 1.15–72.53) and unprotected anal sex with men in the past six months (OR: 6.47; 95% CI: 1.60–26.20) were significant risk factors (Table 3) (Appendix Table 2).

**Table 3:** Factors associated with HIV seroconversions in MSM cohort in Guangzhou and Yangzhou, China

	Yangzhou Multivariate analysis			Guangzhou Multivariate analysis		
	
Factors	*n*	OR (95% CI)	*p*	*n*	OR (95% CI)	*p*
Marital status						
Single	1 (70)	1		7 (147)	–	
Married	10 (114)	0.63 (1.29–53.00)	0.074	4 (34)	–	
Divorced	0 (7)	–		1 (7)		
Anal sex with men in the past 6 months						
Yes	7 (117)	–		11 (107)	9.16 (1.15–72.53)	0.036
No	4 (74)	–		1 (82)	1	
Condom use during last anal sex with a man						
Yes	2 (39)	–		3 (71)	1	
No	5 (78)	–		8 (36)	6.47 (1.60–26.20)	0.009
Condom use during last sexual encounter with a female partner						
Yes	1 (51)	1		1 (23)	–	
No	9 (55)	9.78 (1.19–80.24)	0.034	2 (27)	–	
Syphilis positive						
Yes	7 (50)	5.13 (1.30–20.20)	0.019	1 (15)	–	
No	4 (141)	1		11 (173)	–	

CI: confidence interval; OR: odd ratio.

## Discussion

This is the first cohort study that intended to investigate HIV seroconversion and associated risk factors in a medium-sized Chinese city. Findings from the baseline survey showed that Yangzhou MSM, compared to Guangzhou MSM, were significantly older, less educated and more likely to seek sex in a bathhouse. Yangzhou MSM were more likely to identify themselves as bisexual or heterosexual, to be married and to have unprotected sex with females. They received fewer sexual health services and were therefore less likely to be diagnosed with an STD in the past 12 months. It may be assumed that poorer economic and cultural conditions and traditional thinking may partly explain those differences. However, no information is available to explore these hypotheses, and further investigation is needed.

Compared with MSM who live in Guangzhou and other large-sized cities, the HIV prevalence was higher among Yangzhou MSM. For example, a cross-sectional survey showed that the HIV prevalence was 11.1% among Chengdu MSM in 2008 [13] and 8.0% among Beijing MSM in 2011 [1]. A nationwide Chinese surveillance programme, including 105 sentinel sites, showed that a total of 2343 people were positive for the HIV antibody and demonstrated an overall HIV prevalence of 6.3% among MSM in 2011 [14].

Our results showed that Yangzhou MSM had a higher prevalence of syphilis than Guangzhou MSM. The high prevalence of syphilis in this study was consistent with the percentages found in other reports on MSM populations from different cities [15–22], including 13.5% in Shanghai [23], 11.3% in Harbin [16] and 19.1% in Shenzhen [24]. In bivariate and multivariate analyses, syphilis positivity was associated with HIV infection in this study for both Yangzhou MSM and Guangzhou MSM. The high prevalence of syphilis among MSM means that a large number of MSM are engaging in sexual behaviours that place them at risk for both syphilis and HIV infections. The biological mechanisms through which syphilis increases the risk of HIV infection include syphilitic ulcers that ease the passage of HIV; local inflammation and gathering of CD4+ cells, which increase the possibility of HIV transmission; and activated host immunologic response, which enhances HIV replication [20].

Furthermore, in multivariate analysis, older age (>40 years) was another risk factor of HIV infection in both samples. This finding is consistent with previous studies that showed older MSM to be more at risk for HIV infection than young MSM [25–31]. Many older MSM were unaware of high-risk sexual behaviours, or they thought that their behaviours were of no risk. A study showed that the knowledge level regarding the specific behaviours leading to the transmission of HIV among older adults was significantly lower than among younger adults [31]. Without HIV/AIDS educational and prevention programmes tailored to older audiences, it is likely that many older adults are unwilling to attend or accept HIV education, prevention and intervention activities. Therefore, older MSM perceive themselves to be at low risk for HIV infection, which leads them to have a greater chance of becoming infected with HIV.

Probably due to sociocultural pressure, about 59.0% of Yangzhou MSM were in a marriage. Among those married MSM, 80.3% were bisexual or heterosexual men who were more likely to have sex with females than homosexual MSM [17,32,33], and 75.0% had sex with females in the past six months. However, only 95 among 141 married MSM having sex with females were living with their wives, which means that 32.0% married MSM may have had sex with females other than their wives in the past six months. Meanwhile, they were not likely to have used a condom during their last sexual encounter with the female partner. Consistent with findings for other populations, having unprotected sex outside marriage is a risk behaviour that could increase the HIV spread from man to woman [34–36]. Our results also indicated that married MSM were more likely to become syphilis positive, which may put married MSM at higher risk for HIV seroconversion as syphilis could be a cofactor in HIV transmission [37–41] (Appendix Table 4). Finally, consistent with other studies [42–47], unprotected anal sex with men is related to HIV infection and seroconversion, and it places MSM at increased risk for acquiring and transmitting HIV.

This study first evaluated and compared the HIV incidence density among Yangzhou and Guangzhou MSM utilizing a prospective cohort study design. The HIV incidence density among the two groups (6.78 and 5.77/100 PY) was moderately high, in comparison with that in Beijing (2.6/100 PY), Shenyang (5.4/100 PY) and Nanjing (5.1/100 PY). This rate was also higher than those of other large cities like Paris (3.8/100 PY) [48] and Bangkok (5.9/100 PY) [49]. The higher HIV incidence of Yangzhou MSM may lead to rapid spread of HIV infection among this population. Being married, having female sex partners in the past 12 months and having unprotected sex with female partners in the past six months were risk factors for HIV incidence. Interventions aiming to reduce these risk factors may contribute to alleviating HIV infection in Yangzhou MSM.

There are several limitations that should be considered when interpreting the results of this study. First, snowball sampling is a non-probability sampling technique and has biases, so the representativeness of participants may be limited. Second, we relied on uncorroborated self-reports of sexual behaviours, as is the case in most survey research. The participants may either underreport or over-report risk behaviours, even though every effort (e.g. keeping confidentiality and protecting privacy) was made to minimize this phenomenon. The reliability and validity of self-reported information have also been critiqued by other researchers. Outmigration was an additional problem in our study as only 70.5% of MSM participants in Yangzhou and 65.74% in Guangzhou could be followed during the 12-month period. Therefore, the study estimates may not accurately represent the true HIV and syphilis incidence in the entire population of Yangzhou MSM and Guangzhou MSM, and more effective methods are needed to increase the retention rate for a prospective cohort of MSM (Appendix Table 3). To better characterize the HIV and syphilis epidemic in the future, measures should be taken that improve retention rates and eliminate barriers to participation. How to best improve retention in this at-risk population has not been addressed well in previously published studies and remains a vexing issue for the field.

## Conclusions

From our study, we were able to ascertain estimates of the prevalence and incidence of HIV in MSM and examine the risk factors contributing to HIV infection in a midsized Chinese city. Higher HIV prevalence and incidence, as well as significant differences in demographic characteristics and sexual behaviours, were found in Yangzhou MSM compared to Guangzhou MSM. Such findings indicate that the HIV epidemic among the MSM population of medium-sized cites in China may be more serious than that in large-sized cities. Further studies in MSM from medium-sized cities and HIV preventive programmes targeting relevant risk factors are needed to reduce the spread of the HIV epidemic among MSM in China.

## Appendix

**Table 1:** Factors associated with HIV prevalence at baseline screening among MSM in Yangzhou and Guangzhou, China

	Yangzhou					Guangzhou				
	
	Bivariate analysis			Multivariate analysis		Bivariate analysis			Multivariate analysis	
	
Factors	*n*	OR (95% CI)	*p*	OR (95% CI)	*p*	*n*	OR (95% CI)	*p*	OR (95% CI)	*p*
Age (years)										
20–40	21 (209)	1		1		16 (281)	1		–	
>40	25 (103)	2.09 (1.50–2.90)	<0.001	4.34 (2.25–8.36)	<0.001	3 (18)	0.30 (0.08–1.15)	0.096	–	
Education level										
Middle school or lower	20 (110)	1.23 (0.55–2.75)	0.311	–		2 (26)	1.35 (0.28–6.38)	0.339	–	
High school	15 (135)	0.69 (0.30–1.60)	0.198	–		5 (73)	1.19 (0.40–3.50)	0.369	–	
College or higher	11 (72)	1				12 (206)	1		–	
Marital status										
Single	9 (118)	1		1		9 (239)	1		1	
Married	36 (187)	2.88 (1.33–6.24)	0.002	2.88 (1.33–6.24)	0.007	8 (56)	3.98 (1.49–10.61)	0.005	13.48 (2.06–88.18)	0.007
Divorced	1 (12)	1.10 (0.12–9.51)	0.432	–		2 (10)	5.97 (1.12–31.86)	0.040	5.84 (1.91–17.80)	0.002
Sexual orientation										
Homosexual	26 (134)	1		–		9 (171)	1		–	
Bisexual or heterosexual	20 (183)	1.05 (0.56–1.99)	0.872	–		10 (134)	145 (0.57–368)	0.432	–	
Commercial sex in the past 6 months										
Yes	5 (29)	1.31 (0.47–3.65)	0.600	–		3 (14)	4.69 (1.19–18.49)	0.027	2.01 (0.38–10.56)	0.406
No	41 (288)	1				16 (291)	1		1	
Condom use during last commercial sex encounter										
Yes	2 (17)	1		–		1 (8)	1		–	
No	3 (12)	0.35 (0.05–2.58)	0.307	–		2 (6)	0.60 (0.04–8.73)	0.708	–	
Anal sex with a man in the past 6 months										
Yes	30 (202)	1.07 (0.56–2.07)	0.820	–		17 (177)	6.69 (1.52–29.51)	0.012	9.03 (1.85–44.10)	0.007
No	16 (115)	1		–		2 (128)	1		1	
Condom use during last anal sex with a man										
Yes	21 (40)	1		–		5 (110)	1		1	
No	9 (62)	1.03 (0.44–2.42)	1.000	–		12 (67)	4.58 (1.54–13.67)	0.002	4.93 (1.65–4.69)	0.004
Sex with female partners in the past 6 months										
Yes	36 (188)	2.81 (1.34–5.91)	0.005	2.50 (1.14–5.48)	0.022	3 (52)	0.91 (0.25–3.23)	0.880	–	
No	10 (129)	1				16 (253)	1		–	
Condom use during last sexual encounter with a female partner										
Yes	3 (56)	1		1		1 (14)	1		–	
No	33 (132)	5.88 (1.72–20.10)	0.001	5.89 (1.73–20.11)	0.005	2 (38)	0.72 (0.06–8.65)	0.797	–	
STD-associated symptom in the past 12 months										
Yes	4 (28)	1.07 (0.36–3.13)	0.899	–		3 (39)	1.30 (0.36–4.69)	0.331	–	
No	42 (289)	1		–		16 (266)	1		–	
Diagnosed with an STD in the past 12 months										
Yes	6 (30)	1.09 (0.91–1.31)	0.320	–		9 (82)	2.84 (1.11–7.26)	0.018	2.06 (0.67–6.33)	0.205
No	40 (287)	1				10 (223)	1		1	
Received sexual health services in the past 12 months										
Yes	26 (175)	1		–		16 (265)	1		–	
No	20 (142)	0.94 (0.50–1.76)	0.846	–		3 (40)	1.34 (0.37–4.83)	0.315	–	
Syphilis positive										
Yes	26 (98)	3.59 (1.89–6.83)	<0.001	3.47 (1.76–6.84)	<0.001	5 (20)	6.45 (2.05–20.29)	0.004	6.29 (1.63–24.29)	0.008
No	20 (219)	1		1		14 (285)	1		1	

CI: confidence interval; OR: odd ratio; STD: sexually transmitted disease.

**Table 2:** Factors associated with HIV seroconversions in MSM cohort in Guangzhou and Yangzhou, China

	Yangzhou					Guangzhou				
	
	Bivariate analysis			Multivariate analysis		Bivariate analysis			Multivariate analysis	
	
Factors	*n*	OR (95% CI)	*p*	OR (95% CI)	*p*	*n*	OR (95% CI)	*p*	OR (95% CI)	*p*
Age (years)										
20–40	5 (134)	1		1		11 (176)	1		–	
>40	6 (57)	3.03 (0.88–10.38)	0.044	0.57 (0.29–1.12)	0.107	1 (12)	1.50 (0.17–12.80)	0.527	–	
Education level										
Middle school or lower	7 (66)	3.44 (0.40–29.22)	0.430	–		2 (15)	2.68 (0.50–14.27)		–	
High school	3 (86)	1.15 (0.11–11.53)	1.000	–		3 (45)	1.24 (0.31–5.03)		–	
College or higher	1 (39)	1				7 (128)	1		–	
Marital status										
Single	1 (70)	1		1		7 (147)	1		–	
Married	10 (114)	0.15 (0.02–1.20)	0.053	0.63 (0.29–53.00)	0.074	4 (34)	2.67 (0.73–9.69)	0.128	–	
Divorced	0 (7)	–		–		1 (7)	3.33 (0.35–31.59)	0.316		
Commercial sex in the past 6 months										
Yes	1 (9)	2.15 (0.24–18.91)	0.420	–		1 (8)	2.19 (0.25–19.46)	0.416	–	
No	10 (182)	1		–		11 (180)	1		–	
Condom use during last commercial sex encounter										
Yes	1 (5)	1	–			1 (6)	1		–	
No	0 (4)	–	–			0 (2)	–		–	
Anal sex with a man in the past 6 months										
Yes	7 (117)	1.11 (0.31–3.94)	1.000	–		11 (107)	9.28 (1.17–73.43)	0.013	9.16 (1.15–72.53)	0.036
No	4 (74)	1		–		1 (82)	1		1	
Condom use during last anal sex with a man										
Yes	2 (39)	1		–		3 (71)	1		1	
No	5 (78)	1.31 (0.24–7.12)	0.747	–		8 (36)	0.15 (0.04–0.62)	0.006	6.47 (1.60–26.20)	0.009
Sex with female partners in the past 6 months										
Yes	10 (106)	8.75 (1.09–69.78)	0.024	4.47 (0.53–37.75)	0.168	3 (50)	0.88 (0.23–3.39)	1.000	–	
No	1 (85)	1		1		9 (133)	1		–	
Condom use during last sexual encounter with a female partner										
Yes	1 (51)	1		1		1 (23)	1		–	
No	9 (55)	11.11 (1.36–90.25)	0.008	9.78 (1.19–80.24)	0.034	2 (27)	1.76 (0.14–20.76)	1.000	–	
STD-associated symptom in the past 12 months										
Yes	1 (19)	0.90 (0.11–7.44)	0.922			2 (28)	1.15 (0.24–5.57)	0.694	–	
No	10 (172)	1				10 (160)	1		–	
Diagnosed with an STD in the past 12 months										
Yes	1 (17)	1.05 (0.72–1.54)	0.793	–		3 (51)	0.88 (0.23–3.42)	1.000	–	
No	10 (174)	1		–		9 (137)	1		–	
Syphilis positive										
Yes	7 (50)	5.57 (1.55–19.96)	0.008	5.13 (1.30–20.20)	0.019	1 (15)	1.05 (0.12–8.75)	1.000	–	
No	4 (141)	1		1		11 (173)	1		–	

CI: confidence interval; OR: odd ratio; STD: sexually transmitted disease.

**Table 3:** Comparison of demographic characteristics between retained MSM cohort and those lost to follow-up in Yangzhou and Guangzhou MSM, China

	Yangzhou				Guangzhou			
	
Characteristics	Retained MSM *N* (%)	Dropped MSM *N* (%)	*χ* ^2^	*p*	Retained MSM *N* (%)	Dropped MSM *N* (%)	*χ* ^2^	*p*
Age (years)	39.14±9.01*	36.56±10.53	1.92	0.067	23.32±5.98*	21.73±7.45	1.82	0.081
Education level			1.68	0.429			0.12	0.940
Middle school or lower	66 (34.6)	24 (30.0)			15 (8.0)	11 (11.2)		
High school	86 (45.0)	34 (42.5)			45 (23.9)	23 (23.5)		
College or higher	39 (20.4)	22 (27.5)			128 (68.1)	66 (67.3)		
Marital status			4.12	0.127			2.57	0.275
Single	70 (36.6)	39 (48.8)			147 (78.2)	83 (84.7)		
Married	114 (59.7)	37 (46.2)			34 (18.1)	14 (14.3)		
Divorced	7 (3.7)	4 (5.0)			7 (3.7)	1 (1.0)		
Ethnicity							5.84	0.015
Han	191 (100.0)	74 (92.5)	–	–	185 (98.4)	90 (91.8)		
Other	0 (0.0)	6 (7.5)			3 (1.6)	8 (8.2)		
Venue where sex is found			4.25	0.372			–	–
Bathhouse	108 (56.5)	43 (53.8)			0 (0.0)	5 (5.1)		
Bar	31 (16.3)	15 (18.7)			1 (2.6)	7 (7.1)		
Internet	36 (18.8)	20 (25.0)			160 (85.1)	64 (65.3)		
Other	16 (8.4)	2 (2.5)			27 (14.3)	22 (22.4)		
Sex orientation			6.45	0.011			1.57	0.209
Homosexual	108 (56.5)	31 (38.8)			101 (53.7)	61 (62.2)		
Bisexual or heterosexual	83 (43.5)	49 (61.2)			87 (46.3)	37 (37.8)		
Commercial sex in the past 6 months			2.56	0.109			0.03	0.861
Yes	13 (6.8)	11 (13.8)			7 (3.7)	4 (4.1)		
No	178 (93.2)	69 (86.2)			181 (96.3)	94 (95.9)		
Condom use during last commercial sex encounter			0.20	0.654			0.50	0.477
Yes	10 (62.5)	5 (62.5)			5 (71.4)	2 (50.0)		
No	6 (37.5)	3 (37.5)			2 (28.6)	2 (50.0)		
Anal sex with men in the past 6 months			0.82	0.365			0.85	0.356
Yes	125 (65.4)	47 (58.8)			101 (53.7)	59 (62.2)		
No	66 (34.6)	33 (41.2)			87 (46.3)	39 (39.8)		
Condom use during last anal sex with a man			0.04	0.837			1.75	0.195
Yes	11 (28.2)	4 (13.8)			68 (61.8)	37 (74.0)		
No	28 (71.8)	25 (86.2)			42 (38.2)	13 (26.0)		
Sex with female partners in the past 6 months			3.16	0.075			2.01	0.156
Yes	100 (52.4)	52 (65.0)			37 (19.7)	12 (12.2)		
No	91 (47.6)	28 (35.0)			151 (80.3)	86 (87.8)		
Condom use during last sexual encounter with a female partner			0.18	0.663			0.54	0.463
Yes	36 (33.3)	17 (38.6)			5 (20.0)	8 (33.3)		
No	72 (66.7)	27 (61.4)			20 (80.0)	16 (66.7)		
STD-associated symptom in the past 12 months			2.56	0.109			0.66	0.416
Yes	13 (6.8)	11 (13.8)			21 (11.2)	15 (15.7)		
No	178 (93.2)	69 (86.2)			167 (88.8)	83 (84.7)		
Diagnosed with an STD in the past 12 months			0.44	0.507			2.48	0.115
Yes	15 (7.9)	9 (11.3)			54 (28.7)	19 (19.4)		
No	176 (92.1)	71 (88.7)			134 (71.3)	79 (80.6)		
Received sexual health services in the past 12 months			1.46	0.226			2.01	0.155
Yes	100 (52.4)	49 (61.3)			168 (89.4)	81 (82.6)		
No	91 (47.6)	31 (38.8)			20 (10.6)	17 (17.4)		
Syphilis positive			37.2	<0.001			5.93	0.014
Yes	30 (15.7)	42 (52.5)			5 (2.7)	10 (10.2)		
No	161 (84.3)	38 (47.5)			183 (97.3)	88 (89.8)		

* Independent samples *t-*test.

STD: sexually transmitted disease.

**Table 4:** Comparison of baseline demographic characteristics among married MSM and unmarried MSM in Yangzhou

	Married		Unmarried			
			
Characteristic	*n*	%	*n*	%	Adjusted *χ* ^2^	*p*
Age (years)					–	–
<20	–	–	5	3.8		
20–40	102	54.5	107	82.3		
>40	85	45.5	18	13.8		
Education level					29.52	<0.001
Middle school or lower	81	43.3	29	22.3		
High school	82	43.9	53	40.7		
College or higher	24	12.8	48	37.0		
Marital status					–	–
Alone	34	18.2	106	81.5		
Wife	120	64.2	–	–		
Other	33	17.6	24	18.5		
Sexual orientation					29.63	<0.001
Homosexual	55	29.4	79	60.8		
Bisexual or heterosexual	132	70.6	51	39.2		
Venue where sex is found					37.90	<0.001
Bathhouse	125	66.8	45	34.7		
Bar	35	18.7	31	23.8		
Internet	21	11.2	41	31.5		
Other	6	3.2	13	10.0		
Commercial sex in the past 6 months					17.65	<0.001
Yes	6	3.2	23	17.7		
No	181	96.8	107	82.3		
Condom use during last commercial sex encounter					0.23	0.630
Yes	3	50.0	14	60.9		
No	3	50.0	9	39.1		
Anal sex with men in the past 6 months					2.50	0.114
Yes	112	59.9	90	69.2		
No	75	40.1	40	30.8		
Condom use during last anal sex with a man					3.53	0.060
Yes	71	63.4	69	76.7		
No	41	36.6	21	23.3		
Sex with female partners in the past 6 months					47.33	<0.001
Yes	141	75.4	47	36.2		
No	46	24.6	83	63.8		
Condom use during last sexual encounter with a female partner					0.31	0.581
Yes	40	28.3	16	34.0		
No	101	71.7	31	65.9		
Syphilis positive					6.97	0.008
Yes	69	36.9	29	22.3		
No	118	63.1	101	77.7		
